# How Weight Affects the Perceived Spacing between the Thumb and Fingers during Grasping

**DOI:** 10.1371/journal.pone.0127983

**Published:** 2015-05-21

**Authors:** Annie A. Butler, Martin E. Héroux, Simon C. Gandevia

**Affiliations:** Neuroscience Research Australia and University of New South Wales, Sydney, Australia; Birkbeck, University of London, UNITED KINGDOM

## Abstract

We know much about mechanisms determining the perceived size and weight of lifted objects, but little about how these properties of size and weight affect the body representation (e.g. grasp aperture of the hand). Without vision, subjects (n = 16) estimated spacing between fingers and thumb (*perceived grasp aperture*) while lifting canisters of the same width (6.6cm) but varied weights (300, 600, 900, and 1200 g). Lifts were performed by movement of either the wrist, elbow or shoulder to examine whether lifting with different muscle groups affects the judgement of grasp aperture. Results for perceived grasp aperture were compared with changes in perceived weight of objects of different sizes (5.2, 6.6, and 10 cm) but the same weight (600 g). When canisters of the same width but different weights were lifted, perceived grasp aperture decreased 4.8% [2.2 ‒ 7.4] (mean [95% CI]; *P* < 0.001) from the lightest to the heaviest canister, no matter how they were lifted. For objects of the same weight but different widths, perceived weight decreased 42.3% [38.2 ‒ 46.4] from narrowest to widest (*P *< 0.001), as expected from the size-weight illusion. Thus, despite a highly distorted perception of the weight of objects based on their size, we conclude that proprioceptive afferents maintain a reasonably stable perception of the aperture of the grasping hand over a wide range of object weights. Given the small magnitude of this ‘weight-grasp aperture’ illusion, we propose the brain has access to a relatively stable ‘perceptual ruler’ to aid the manipulation of different objects.

## Introduction

Grasping is an important human sensorimotor function which is finely controlled. Our sensorimotor system easily incorporates novel grasped objects with a wide range of sizes and weights into our working ‘space’ [1] and we seamlessly alter digit placement and force on a grasped object depending on the task [2–4]. The ability to grasp, lift and manipulate objects involves complex movements of many joints and contraction of multiple muscles (e.g. [5]) and can be performed accurately even without vision [6, 7]. An everyday action, like lifting a cup, requires a motor plan that relies on an accurate body ‘representation’ of where our hand and fingers are in space and this information is continuously available via sensory inputs from skin, joint and muscle receptors (e.g. [8–10], for review see [11–13]). However, none of these receptors directly encode the distance between parts of the body. For this, afferent information needs to be interpreted in the context of a central ‘representation’ of the body [14, 15]. This central representation and how afferent information is interpreted by the central nervous system are highly malleable and artificial changes in sensory inputs can be misinterpreted by the brain as genuine physiological signals. This can produce misjudgements of the location of the hand and fingers [15–18]. For example, vibration of grasping muscles [19], electrical stimulation of their afferents [20], and stretching of nearby skin [21] disturb the perceived configuration of the hand.

Grasping is influenced powerfully by prior experience and learning based on a lifetime of manipulation of objects [3, 22]. For example, when the weight of a lifted object is initially unknown, the motor system quickly learns to control the forces required to lift it [3, 23]. Also, the expectations that are built on this learning have dramatic effects on weight perception. When reaching for an object, grasp aperture is formed and scaled [24–26]. After touching and grasping it, but prior to lifting, cutaneous receptors in the pads of the digits and proprioceptive afferents that signal the configuration of the hand provide information about some object properties (e.g. size and texture), which create an expectation about other properties (e.g. weight, density) that underpin the motor plan to lift it [3, 23, 27–31]. Once lifted, the object’s perceived weight is determined by peripheral inputs and the level of voluntary command ([32–34], see also [35]) although the strong influence of expectation on perceived weight persists.

The size-weight illusion is a familiar example of how the expectations that result from prior experience bias how we interpret sensory inputs: for two objects of the same weight but different size, the smaller (expected to be light) is perceived as much heavier [36]. The magnitude of the size-weight illusion can diminish with training but this takes several days of lifting (see [27, 37]). Even when vision is unavailable, somatosensory signals alone produce an equally robust size-weight illusion [38]. Similar to this illusion, Usnadze [39] exposed a ‘weight-size illusion’: when objects were placed on the hand under passive conditions, heavier objects (expected to be large) were perceived as smaller than lighter ones. Using different reporting methods, ranges of weights, object shapes and lifting methods, other researchers have shown that when objects are lifted, heavier objects are perceived as slightly larger when lifted [40, 41]. However, these studies do not compare the magnitude of the size-weight illusion with that of the weight-size illusion. Also, it is unknown whether object properties and the expectations associated with these properties [31, 38, 42] affect how the brain perceives the hand that is performing the task. Specifically, does our estimate of the grasp aperture of the hand (a proprioceptive judgement of the spacing between the fingers and thumb) alter with the size and weight of a lifted object? This is important because sensory deficits, including deficits in proprioceptive inputs, can impair grasping (e.g. [43–45]). Furthermore, many clinical conditions are characterised by a defective body representation [46] which in some conditions, including schizophrenia and anerexoria nervosa, can influence how individuals perceive the properties of everyday objects [47–50].

Our primary hypothesis was that the perceived grasp aperture of the hand is affected by the weight of a lifted object to a similar extent as the size of an object affects its perceived weight—that is the classic size-weight illusion. Specifically, we hypothesised when heavier objects are lifted, grasp aperture will be perceived as narrower compared to when lighter objects of the same size are lifted.

Although the hand and digits are always involved when we grasp an object, we can lift an object, and thus estimate its weight, with muscles in the wrist, arm and shoulder. As the weaker more distal muscles of the upper limb have greater motor (muscle) noise than stronger proximal muscles [51], we hypothesised that the influence of an object’s weight on grasp aperture judgements would differ when different muscle groups are used to lift objects. Thus, we examined whether the muscle groups used to lift the objects affect judgement of the grasp aperture of the hand. In addition, we compared the magnitude of the influence of weight on perceived grasp aperture with the magnitude of the size weight-illusion. For all experiments, we controlled the thixotropic state of the muscle which has a potent effect on proprioceptive judgements via muscle spindle signals [12, 52]. Preliminary results have been presented at Physiology 2014 [53].

## Materials and Methods

### Subjects

A total of 18 healthy subjects participated in the studies (11 female, range 25–58 years). Each of the experiments (1 and 2) included 16 subjects. Fourteen subjects participated in both experiments 1 and 2. All were naïve to the exact hypotheses tested. Subjects gave written informed consent, and the experimental procedures were in accordance with the Declaration of Helsinki (2008) and approved by the University of New South Wales Human Research Ethics Committee (HC11442).

### General experimental procedures

**Fig 1:**
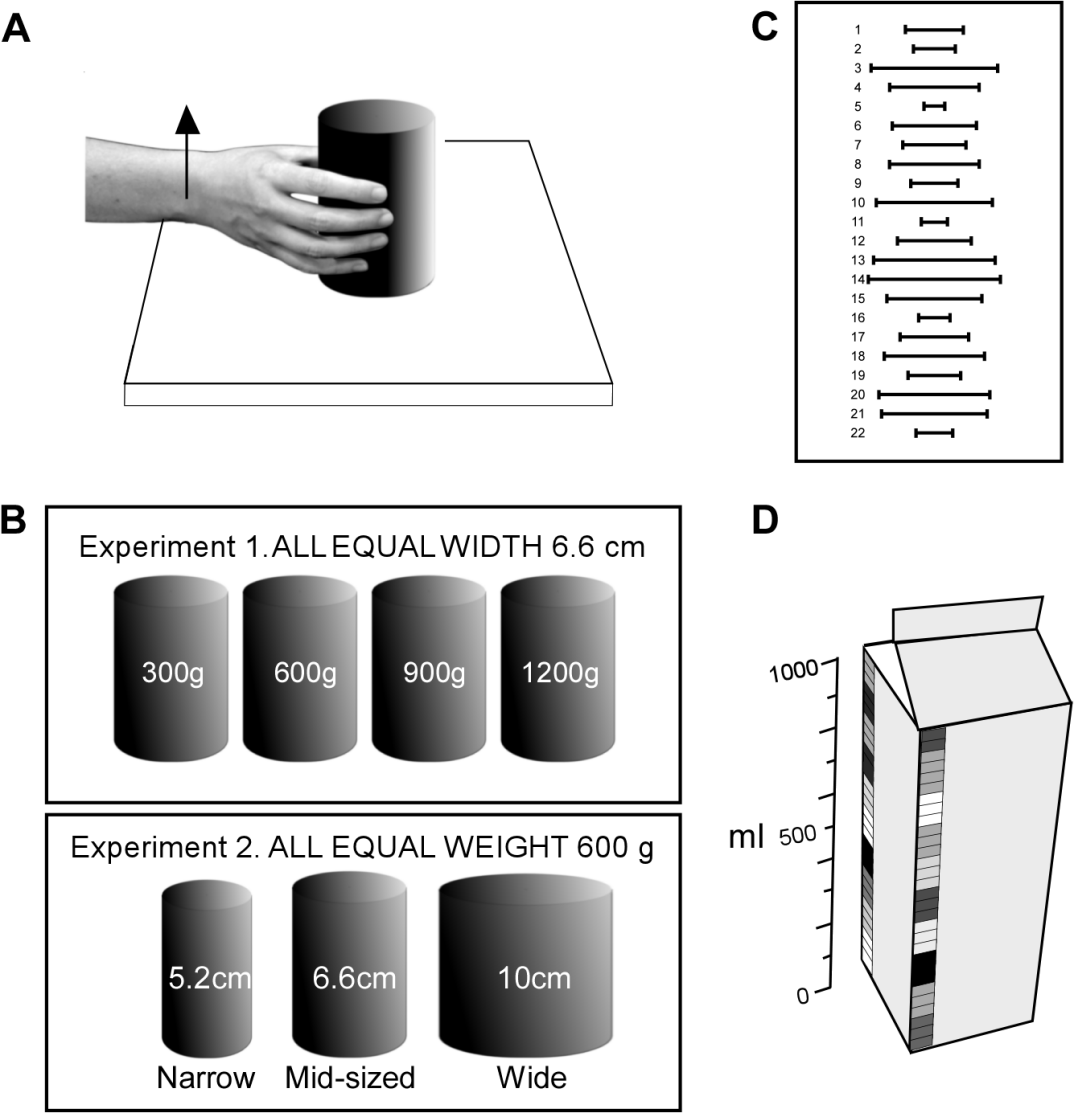
Experimental set-up. A, in both experiments 1 and 2, subjects grasped and lifted a range of standard cylindrical canisters of different weights and widths. The canisters were lifted with the right hand with movement at the wrist, elbow or shoulder. Throughout all experiments, the canisters and the subject’s arm were screened from view. B, shows the canisters of different dimensions lifted in both experiments. For experiment 1, the *mid-sized* test canisters (width 6.6 cm) were used to measure perceived horizontal spacing between the fingers and thumb (*perceived grasp aperture*) during a lift. These four test canisters ranged in weight from 300g–1200g. The *narrow* and *wide* canisters from experiment 2 each weighed 600g and were used as distractors (see Methods). For experiment 2, the test canisters included the *narrow*, *mid-sized* and *wide* canisters which each weighed 600g and ranged in size from 5.2cm–10cm. During the lift subjects reported the weight, they perceived to be lifting. Two *mid-sized* canisters from experiment 1 of 300 g and 900 g were used as distractors (see Methods). C, in experiment 1, subjects reported their perceived grasp aperture using an A3 sized visual chart with 22 numbered horizontal lines of different lengths that represented the grasp aperture of the hand. D, in experiment 2, subjects reported the weight of milk they perceived to be lifting using a coloured vertical scale marked on a one-litre carton with ten 1.9-cm coloured increments, each divided into upper, middle and lower portions for each colour. Each portion represented 33.3 g.

Without vision, subjects grasped and lifted a range of cylindrical canisters of different sizes and weights with their right hand and made judgements on the perceived horizontal spacing between their fingers and thumb (*perceived grasp aperture*; experiment 1) or perceived weight of the lifted object (experiment 2). Fig 1 shows the experimental set-up. Subjects sat comfortably with their right forearm and hand resting on a table, both of which were concealed from view by a screen. With the subject’s arm in a relaxed position, the experimenter placed each canister between the subject’s fingers and thumb. All fingers contacted the canister and were opposed to the thumb, as if lifting a glass. Subjects were told to vertically lift the canister ~3 cm above the table for ~5 s, and not to feel or explore other aspects of the canister with their fingers.

The number of lifts and canister weights were selected to minimise fatigue. Between trials subjects made one brief voluntary contraction of the fingers and thumb to form a fist and then relaxed the hand on the table. This manoeuvre was done deliberately to leave muscle spindle afferents in a similar initial state [12, 52]. Before each experiment, subjects were familiarised with the lifting techniques and reporting methods.

### Experiment 1. Does the weight of an unseen lifted object affect the perceived grasp aperture of the hand?

This experiment was designed to examine whether the perceived horizontal spacing between the fingers and thumb (*perceived grasp aperture*) changes if canisters of the same size but different weights are lifted. Further, we examined whether the mode of lifting altered perceived grasp aperture (see below).

In each experimental session there were six different canisters. There were four test canisters of equal width (6.6 cm width, *mid-sized*) but varied weight (300, 600, 900 and 1200 g; Fig 1B), and two additional distractor canisters weighing 600 g, one *narrow* (5.2 cm width) and one *wide* (10 cm width). The two additional 600-g canisters were included as ‘distractors’ to vary canister width during the trials. All canisters were made of steel and had a uniform texture. Small lead weights were distributed evenly throughout the volume of the canisters and gaps were filled with Styrofoam.

The six canisters were presented in a pseudo-random order. There were 6 lifts for each test canister (300, 600, 900 and 1200 g; *mid-sized*). Lifts of the *narrow* and *wide* canisters (distractors) were randomly distributed throughout the trials. The lift trials were performed in three ways, each on three separate days. Subjects grasped and lifted the canisters with (i) wrist movement only with the forearm resting on the table (i.e. wrist abduction and adduction), (ii) elbow movement only with the wrist joint held steady (i.e. elbow flexion and extension) and (iii) shoulder movement only, lifting the forearm and elbow clear of the table with the wrist and elbow joints held steady (i.e. shoulder flexion and extension). These movements use different muscle groups and represent a range of lifting methods. The same 16 subjects performed each of the three types of lift. Each experimental session took ~40 minutes.

During each lift subjects reported their perceived grasp aperture from a selection of 22 horizontal lines in the shape of error bars (├──┤)that ranged in width from 2 to 12.5 cm in 0.5 cm increments printed on an A3 sheet of paper (Fig 1C). The horizontal lines were centred and numbered from 1 to 22. Twenty different charts were used to report grasp aperture. On each sheet the horizontal lines were arranged in a different order. During each lift a sheet was presented to the subjects at eye level directly above their test hand. The subject was asked to "*select the line which best matches the horizontal spacing between your fingers and thumb*?” If necessary, subjects could select an aperture midway between two lines on the sheet. This chart reporting method is similar to that devised by Gandevia and Phegan [54] and used by others (e.g. [17, 55]). This method also avoids potential problems when reporting with the contralateral hand [4, 56].

### Experiment 2. The effect of canister width on the perceived weight of an unseen lifted object

This experiment was designed to examine how much the perceived weight changes when canisters of the same weight but different sizes are lifted.

Subjects (n = 16) lifted a range of cylindrical canisters that varied in width but had a constant weight (600 g). During each lift they were required to report the perceived weight of the canister as a given volume in a standard one-litre milk carton (7.0 cm width by 19.3 cm height). Following trials of different reporting methods a milk carton was chosen because it is a familiar everyday item that people are accustomed to lift with varied weights. Without vision, subjects used their right hand to grasp the canister and lifted it with wrist movement only (Fig 1A). The three test canisters (600 g) varied in size (width; *narrow* 5.2 cm, *mid-sized* 6.6 cm and *wide* 10 cm; Fig 1B). Each canister was lifted in a pseudo-random order, and was repeated 12 times. Also, two distractor canisters of the same size but different weights (6.6 cm, 300 and 900 g) were presented alternately between each block of three lifts to vary canister weight during the trials.

During each lift, subjects verbally reported the perceived volume (i.e. an index of weight) they were lifting. The one-litre carton sat on a table at eye level directly above the subject’s lifting hand and was fitted with a scale on its side (Fig 1D). The scale was marked with ten 1.9 cm coloured increments, each divided into upper, middle and lower portions for each colour. Each portion represented 33.3 g of liquid. The carton was rotated after each lift to display a new randomly ordered scale of colours. There was no upper limit on the scale and subjects were allowed to report over one litre if required.

Before each test lift subjects lifted a *mid-sized* reference canister (width 6.6 cm) that weighed 1 kg and were told it represented the weight of the contents of a full one-litre carton of milk. Next, subjects lifted the test canister and were asked to report *“relative to the weight of the full carton you just lifted*, *for this canister*, *what level or weight of milk do you think it represents in the carton*?” Pilot experiments did not include the 1 kg reference weight and we found that the perception of weight drifted significantly over time (unpublished observations). Hence, the 1-kg reference weight was added. The duration of the experiment was ~ 45 minutes.

### Data and statistical analysis

Data from the distractor canisters were not used in the main analysis but were used to validate our reporting methods and determine the extent to which subjects could perceive accurately real differences in object width and weight. Linear regression was performed on each subject’s perceived grasp aperture for the 600-g canisters of 3 different widths (5.2, 6.6, 10 cm) and perceived weight for the 6.6-cm canisters of 3 different weights (300, 600, 900 g) to determine the strength of the relationship between actual and perceived grasp aperture and actual and perceived weight.

For each test canister the first trial was excluded and the subsequent five (experiment 1) or eleven trials (experiment 2) were used for analysis. For experiment 1, a two-way repeated measures ANOVA compared perceived grasp aperture for each 6.6-cm canister of different weight (300, 600, 900, 1200 g) and the type of movement used to lift the canister (wrist, elbow or shoulder). Post-hoc tests with Bonferroni correction were used. As there was no effect of the movement type in experiment 1 (see Results), data were pooled across the three movement conditions and expressed as a ratio of the mean perceived grasp aperture across all lifts of the test canisters for each subject. These normalised data were then compared across the four test canisters (6.6 cm width; 300, 600, 900, 1200 g) using a one-way repeated measures ANOVA. For experiment 1, data were normalised as a ratio of the mean perceived grasp aperture for all test trials for each subject (*ratio* = *perceived grasp aperture / mean perceived grasp aperture of all trials)*. For experiment 2, data were normalised as a ratio of the mean perceived weight for all test trials for each subject (*perceived weight / mean perceived weight of all trials*). Data were then compared across the three test canisters (600 g weight; 5.2, 6.6, 10 cm) using a one-way repeated measures ANOVA. In Results, unless indicated, changes across conditions are expressed using these normalised values. The degrees of freedom were corrected using the Greenhouse-Geisser procedure when assumptions of sphericity were not met.

To assess the variability of judgments of grasp aperture when lifting with different movement types, coefficients of variation (SD / mean) were calculated for each subject when lifting the 600-g test canister. The data for the wrist, elbow and shoulder were compared using a one-way repeated measures ANOVA.

The overall magnitude of the illusion was calculated for experiment 1 as *(mean perceived aperture 300-g canister ‒ mean perceived aperture 1200-g canister) / mean perceived aperture 300 g*, and for experiment 2 as *(mean perceived weight 5*.*2-cm canister ‒ mean perceived weight 10-cm canister) / mean perceived weight 5*.*2-cm canister*. The data from the 14 subjects who participated in both experiments were used to investigate the relationship between the strength of the illusion in experiment 1 and experiment 2. For each subject, regression lines were fitted to the perceived grasp aperture across the four canister weights (experiment 1) and the perceived canister weight across the three canister sizes (experiment 2). The slope of these regression lines are a measure of the relative strength of the illusion. Spearman’s rank correlation was then used to assess the relationship between these slopes. To further compare the magnitudes of the illusions of experiment 1 and 2, we compared the changes in perceived grasp aperture for a doubling in canister weight (300g vs. 600 g; experiment 1) to the changes in perceived weight for an approximate doubling in canister width (5.2 cm vs. 10 cm; experiment 2).

Statistical significance was set at *P* < 0.05, and all tests were carried out using SPSS (version 21, SPSS Inc., Chicago IL). Data are presented as mean [95% confidence intervals (CI)] unless otherwise stated.

## Results

### Experiment 1. Does the weight of an unseen lifted object affect the perceived grasp aperture of the hand?

**Fig 2:**
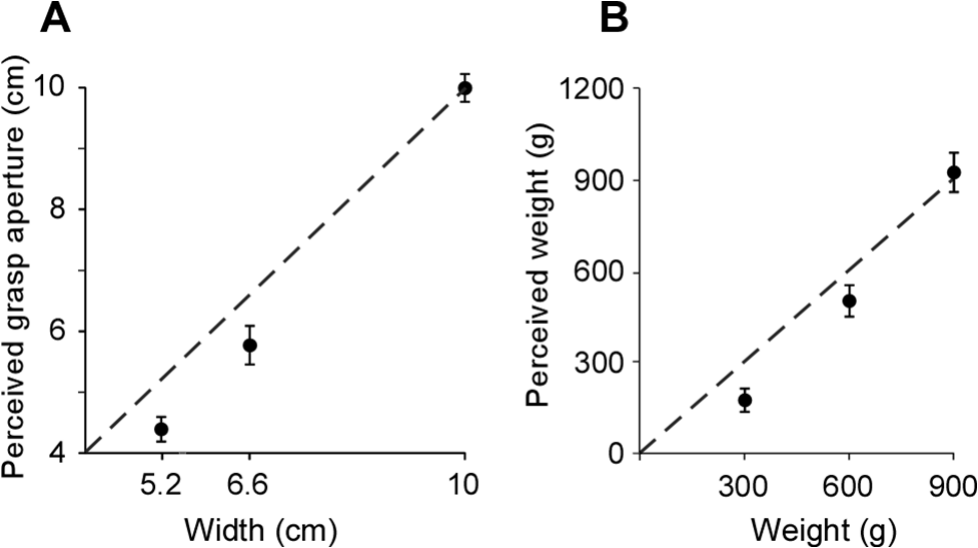
Relationship between actual and perceived grasp aperture (experiment 1), and actual and perceived weight (experiment 2). A, the relationship between actual and perceived grasp aperture for the three canisters of the same weight (600 g) but different width (5.2, 6.6, 10 cm). Data are presented as mean [95%CI]. Actual and perceived grasp aperture were linearly related with a mean *R* ^2^ value of 0.99 [0.99 ‒ 1.00]. The dashed line is the line of identity. On average, grasp aperture was underestimated. B, the relationship between actual and perceived canister weight for the three canisters of the same width (6.6 cm) but different weight (300, 600, 900 g). Actual and perceived weight were linearly related with a mean *R* ^2^ value of 0.98 [0.97 ‒ 0.99]. Data are presented as mean [95% CI] and the dashed line is the line of identity. On average, canister weight was underestimated.

**Fig 3:**
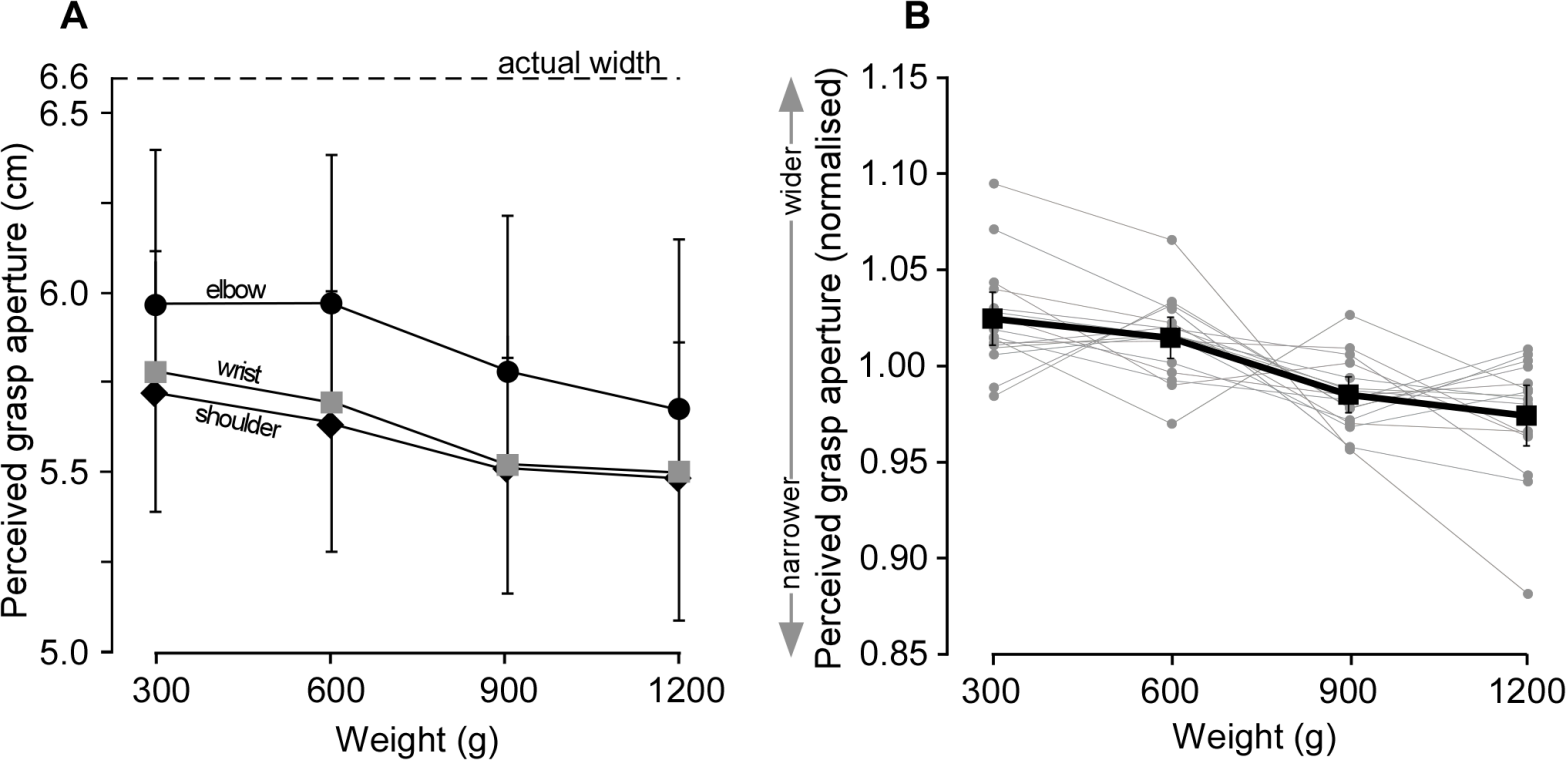
The effect of canister weight on perceived grasp aperture of an unseen lifted object (experiment 1). A, filled symbols represent mean [95% CI] perceived grasp aperture for each of the four test canisters lifted, using movement at the elbow (black circles), wrist (grey squares) and shoulder (black diamonds). The test canisters had the same width (6.6cm) but varied in weight (300–1200 g). Perceived grasp aperture was narrower when heavier canisters were lifted compared to lighter ones (*P*<0.001). Results are similar with movement at the wrist, elbow or shoulder (*P* = 0.16). Dashed horizontal line represents the actual canister width (6.6cm) which was consistently underestimated. B, data are presented as ratios normalised to the mean perceived grasp aperture of all four lifted weights for each subject at each joint (*perceived grasp aperture/mean perceived grasp aperture of all trials*). Individual data are shown as grey circles. Squares show the mean ratio pooled across the three types of movement used to lift the canister [95% CI]. For the pooled data, perceived grasp aperture is significantly narrower (4.8%) when the heavier canisters are lifted compared to the lighter canisters of the same width (*P* < 0.001).

Subjects were able to discriminate between canisters that had the same weight but different widths. There was a strong linear relationship between canister width and perceived grasp aperture (horizontal spacing between the fingers and thumb) when subjects grasped and lifted canisters of the same weight (600 g) but different widths (mean *R* ^*2*^ = 0.99 [0.99 ‒ 1.00]; *P<*0.001; Fig 2A). The mean perceived grasp aperture was 4.39 cm [4.19 ‒ 4.59] for the small 5.2-cm canister, 5.77 cm [5.45 ‒ 6.09] for the 6.6-cm mid-sized test canister, and 9.99 cm [9.76 ‒ 10.22] for the large 10-cm canister. On average, when the test canisters (6.6cm) were lifted, grasp aperture was underestimated by 0.83 cm [0.63 ‒ 1.03] (Fig 3A; *P*<0.001).

The primary finding is that when objects of the same width (6.6 cm) but different weights (300, 600, 900, 1200 g) were lifted, the perceived grasp aperture of the hand decreased as the weight lifted increased. Although perceived grasp aperture was influenced by the weight lifted (*F*_3,45_ = 12.3, *P*< 0.001; Fig 3A), there was no statistically significant effect of movement type (i.e. wrist, elbow or shoulder; *F*_2,30_ = 1.9, *P* = 0.16). The data were therefore pooled and normalised across the three movement conditions (see Methods).

Overall, perceived grasp aperture decreased significantly by 4.8% [2.2 ‒ 7.4] across the range of lifted weights (*F*_1.8, 26.8_ = 10.3, *P*< 0.001; Fig 3B). Post-hoc analysis showed perceived grasp aperture decreased by 4.8% between lifts of 300-g and 1200-g weights, 3.9% between the 300-g and 900-g weights, 2.9% between the 600 g and 900 g weights, and 4% between the 600-g and 1200-g weights (post-hoc, all *P*< 0.05).

The variability in perceived grasp aperture did not change when different movement types were used to lift the 600-g test canister (coefficient of variation: wrist 0.09 [0.07–0.12], elbow 0.07 [0.06–0.09], shoulder 0.08 [0.05–0.11]; *F*_2,30_ = 1.1, *P* = 0.33).

In summary, these results show that the weight of a lifted canister has a small effect on perceived grasp aperture (4.8%). This ‘weight-grasp aperture’ illusion occurs whether canisters are lifted with wrist, elbow or shoulder movement.

### Experiment 2. The effect of canister width on the perceived weight of an unseen lifted object

**Fig 4:**
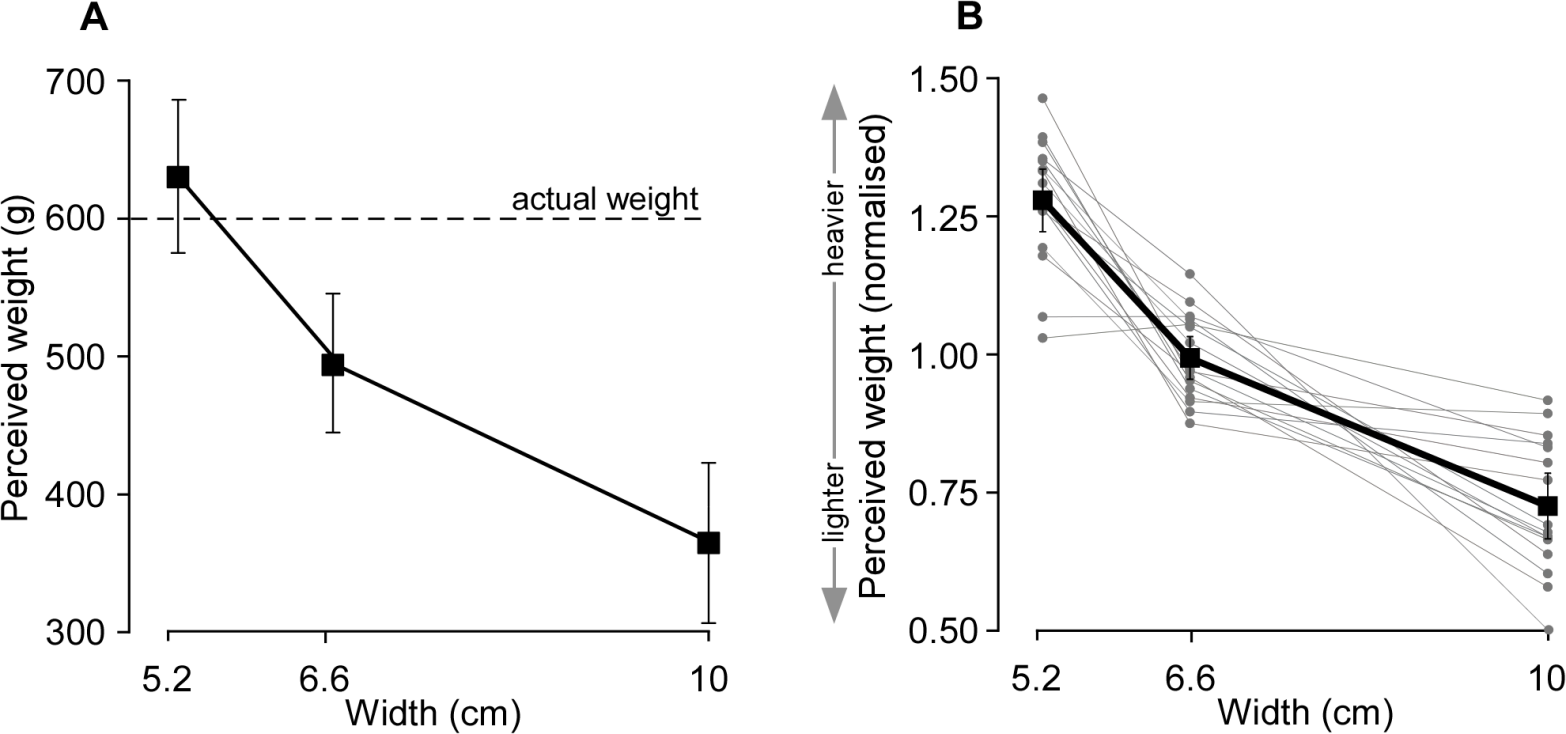
The effect of canister width on perceived weight of an unseen lifted object (experiment 2). A, shows the mean [95% CI] perceived weight of each of the three lifted canisters of the same weight (600 g) with varied widths. The perceived weight differed significantly with changes in canister width. The *narrow* canister felt much heavier than *wide* canisters (P < 0.001). Dashed line represents the actual canister weight (600g). B, shows the mean [95% CI] perceived weight of all trials for each subject normalised and presented as a ratio (*perceived weight / mean perceived weight of all trials;* squares). Individual data are indicated by grey circles. On average, perceived weight decreased by 42.3% from the narrowest to the widest canister although all canisters weighed 600 g (P<0.001).

There was a linear relationship between actual and perceived weight for canisters of the same width (mean *R* ^2^ = 0.98 [0.97 ‒ 0.99]; *P* < 0.001). Subjects were able to distinguish differences between the three canisters of the same width (6.6 cm) but varied weight; 168 g [131 ‒ 205] for the 300-g canister; 494 g [443 ‒ 545] for the 600-g canister; and 915 g [815 ‒ 979] for the 900-g canister (Fig 2B). On average, the weight of the test canisters (600 g), was underestimated by 103 g [54 ‒ 152] (Fig 4A; *P*<0.001).

Although the three canisters weighed 600 g, subjects perceived the *narrow*, *mid-sized* and *wide* canisters (5.2, 6.6 and 10 cm, respectively) as having a different weight. The *narrow* canister had the greatest perceived weight while the *wide* canister had the lowest perceived weight (*F*_2,30_ = 72, *P* < 0.001; Fig 4A). This is expected from the size-weight illusion. Normalised individual data and group results are shown in Fig 4B and indicate that normalised perceived weight decreased by 42.3% [38.2 ‒ 46.4] from the narrowest to the widest canisters (which all weighed 600 g), 22.4% between the *narrow* and *mid-sized* canister and 26.5% between the *mid-sized* and *wide* canisters (*F*_2,30_ = 71, *P* < 0.001; post-hoc, *P*< 0.01; Fig 4B).

### Relationship between the proprioceptive illusions in experiment 1 and 2

Overall, subjects perceived their grasp aperture as widest when lifting the lightest canister (experiment 1) and perceived the weight of the widest canister as lightest (experiment 2). Conversely, subjects perceived their grasp aperture as narrowest when lifting the heaviest canister and perceived the weight of the narrowest canister as heaviest. However, the magnitude of the illusion in experiment 1 was only a tenth of that in experiment 2 (4.8% versus 42.3%) and, for the 14 subjects who completed both experiments, the magnitude of the effect of canister weight on perceived grasp aperture and the effect of canister width on perceived weight were not correlated (Spearman’s *R* = -0.18, *P =* 0.53).

## Discussion

Our main novel result is that the weight of objects that we lift affects how we perceive the grasp aperture of the hand. A four-fold increase in object weight resulted in a ~5% decrease in perceived spacing between the fingers and thumb (grasp aperture). This illusion occurred irrespective of whether the object was lifted with movement restricted to the wrist, partially restricted at the elbow, or unrestricted at the shoulder. This was measured when the thixotropic state of spindles in the grasping muscles was controlled [12, 52]. Importantly and contrary to our initial hypothesis, object weight had only a small effect on perceived grasp aperture compared to the large effect that object size had on perceived weight. Finally, relative to actual values, both the representation of our hand (‘grasp aperture’) and the weight of an external object were underestimated.

Changes in several proprioceptive channels may contribute to altered judgements of grasp aperture. In our experiments, the grasp force used to lift heavier canisters was increased, but the size of canister was constant, so there was no change in joint angles, muscle lengths or in skin stretch around the finger joints. When heavier objects are lifted, there will be increased muscle spindle discharge due to increased motor command and associated fusimotor drive [57, 58]. However, the encoding of length changes by a population of spindle endings does not translate into a ruler-like measure of the length of the muscle-tendon unit [59]. The central nervous system will also use its internal representation of the body to model the predicted sensory consequence of a motor command [60, 61]. In this scenario, the additional spindle activity would be expected by the central nervous system and would not be interpreted as an increase in grasp aperture. Also, the area of contact between the skin and object may increase due to compliance of the digit pads when heavier objects are lifted, and this may cause small changes in joint position. Contact area of the finger pads increases only minimally for forces >1N [62, 63]. As forces in our study exceeded 1N, altered finger pad compression is unlikely to contribute to the decrease in perceived grasp aperture. Furthermore, overall grasp aperture (defined as the spacing between the surface of the fingers and thumb) remains constant irrespective of the amount of pad compression. Finally, skin stretch at proximal sites in the hand could give signals related to thumb and index position and hence provide information on the distance between the pads of the digits [9, 64]. However, when objects of the same size are grasped, there is likely no change in skin stretch.

The perceived aperture of the hand was the same when different muscle groups were used to lift the objects. Greater motor noise accompanies contractions with weaker more distal muscles [51] and may alter the judgement of weight and influence perceived grasp aperture. We found all three lifting movements about the wrist, elbow, and shoulder resulted in a similar decrease in perceived grasp aperture as the weight of the object increased with no difference in the variability of judgements of grasp aperture across the different types of movement.

Based on recent work showing an effect of centrally generated motor commands on perceived joint position [65–70], the perceived narrowing of grasp aperture when lifting heavier objects in our experiment is consistent with a possible effect of central motor command. For example, voluntary isometric ‘efforts’ cause illusory changes in joint position (in the direction of the effort) that are scaled to the amount of effort, and voluntary efforts in the oculomotor system produce large visual illusions [71]. Thus, the increased forces used to lift heavier objects may result in an illusory reduction of grasp aperture. However, when judging object size, the magnitude of the grasp force used to hold an object does not influence its perceived size [72] despite having an influence on its perceived weight [28, 73].

In experiment 1, grasp aperture was perceived as narrower when lifting heavier objects than lighter objects of the same size. Thus, perception of grasp aperture is affected by the weight of an object. This ‘weight-grasp aperture’ illusion is likely driven by the same expectation formed by object properties that occurs in the ‘size-weight’ illusion ([36], see [74] for review]) and the ‘weight-size’ illusion [39]. For the ‘weight-size’ illusion, heavier objects are perceived as smaller than lighter objects when their size is the same, and we hypothesised that this would also be associated with a reduction in perceived grasp aperture. In our study the magnitude of the weight-grasp aperture illusion was small (~5%) compared with the magnitude of the size-weight illusion (~42%). However, the weight range of canisters used in experiment 1 (300g-1200g) was about 25% of the maximal canister weight that subjects could lift (~4kg) whereas, the range of canister widths used in experiment 2 was 4.8cm, ~40% of maximal grasp aperture (~12cm). When these ranges are scaled in order to allow for a comparison, the magnitude of the size-weight illusion remains much larger than the weight-grasp aperture illusion. It is as if proprioceptive signals of grasp aperture reduce the magnitude of the weight-grasp aperture illusion. In support of the predominant role of these proprioceptive signals in human perception, afferent signals of joint position are known to reduce the effect of central signals of motor command on perceived joint position ([67], see also [69]). It is also consistent with the observation that visual illusions have less effect on the perceived size of the hand than the perceived size of other body parts [75]. Thus, the grasping hand can act as a ‘perceptual ruler’ largely unaffected by the weight of the object and how it is lifted.

Some previous results on the weight-size illusion do not fit with our findings. When judging the size of lifted objects, Bergman [40] and Hirsiger and colleagues [41] showed that for objects of the same size but different weight, heavier objects were perceived as larger. However, Hirsiger and colleagues [41] showed an overall small effect of weight on perceived object size, with no effect for the weights used in our experiments (300 g and 600 g). There are also a number of methodological differences between these studies and our own. For example, Hirsiger and colleagues’ experiments were performed with a lighter range of weights. Also, boxes were lifted with the thumb and index finger only and different reporting methods were used. Another important difference is that we controlled the thixotropic state of the muscles to ensure the effect of muscle contraction history was consistent in each trial. Perception can be affected by the history of contraction of the muscles in both the active lifting hand and an ‘indicator’ hand [52, 69]. Finally, we measured perceived aperture of the hand rather than perceived object size (see [40, 41]). It is unknown whether there is a difference between perceived grasp aperture and perceived object size.

While we have shown that object weight affects the perceived aperture of the hand, this small effect is unlikely to affect our grasping ability. When a property of an object (e.g. weight or texture) changes unexpectedly, the motor system adapts quickly to adjust the forces used to lift the object [3, 23, 31]. Furthermore, the motor system adapts to the size-weight illusion within a few lifts, whereas it takes many days of training to overcome the distorted perception of weight [27, 76]. A different example of disparity between perceptual and motor systems shows that visual illusions have a large effect on the perceived size of an object, yet have only a small effect on the accuracy of grasp aperture when reaching for the same object [77–79]. These studies illustrate the fast adaptation of the motor system for lifting but slow adaptation of the perceptual system for the judgement of object size and weight.

The illusions assessed here are easy to appreciate from everyday experience. If a bottle is grasped and lifted at its widest part, then put down and lifted at its neck which is narrower, the bottle will feel much heavier. However, in terms of perceived grasp aperture, if a full bottle is lifted, grasp aperture will feel at most only slightly narrower when it is full, compared to when it is empty, despite a large change in weight of the bottle.

In summary, the perceived spacing between the fingers and thumb decreased when heavier objects of the same size were lifted. The influence of the weight of objects on perceived grasp aperture dependent on which muscle groups were involved in the lift. The effect of this weight-grasp aperture illusion was small compared with the well-known size-weight illusion in which expectation strongly influences the perceived weight of an object. We propose that proprioceptive afferents effectively attenuate the influence of the weight of an object on how we perceive the aperture of the hand. Our findings highlight the relative stability of the representation of the hand, a stable ‘perceptual ruler’ which would underpin skilled interaction between the hand and the external world. The interplay between the perceptual ruler and expectation based on an object’s weight is complex but it may contribute to the difficulties in the control of grasping in people with neurological conditions.
